# A Case of Ocular Syphilis in an HIV-Positive Patient With Penicillin Allergy

**DOI:** 10.7759/cureus.29203

**Published:** 2022-09-15

**Authors:** Merly Cubelo, Odelvys Granela, Rishi Kalia, Francoeur Cadet

**Affiliations:** 1 Dr. Kiran C. Patel College of Osteopathic Medicine, Nova Southeastern University, Fort Lauderdale, USA; 2 Infectious Diseases, AdventHealth Orlando, Orlando, USA

**Keywords:** benzathine penicillin g, syphilis, penicillin allergy, hiv positive, ocular syphilis

## Abstract

Ocular syphilis has a wide range of presentations; however, the most common findings are ophthalmalgia, blurry vision, and erythematous conjunctiva. This report presents a case of ocular syphilis in a human immunodeficiency virus-positive patient with an allergy to penicillin. On presentation, the patient was diagnosed using a fluorescent treponemal antibody absorption test, in addition to rapid plasma reagin. In this case, the patient received alternative treatment with doxycycline prior to penicillin desensitization, with marked improvement of his symptoms.

## Introduction

Syphilis is a sexually transmitted disease caused by the spirochete *Treponema pallidum*. The clinical course of untreated syphilis can progress through four different stages, namely, primary, secondary, latent, and tertiary syphilis. Primary and secondary syphilis commonly occur weeks to months following the initial infection and present with a chancre at the inoculation site and constitutional symptoms with a diffuse rash that classically involves the palms and soles. These stages can be asymptomatic. Patients who remain untreated during early syphilitic states go on to develop latent syphilis [1,2]. The clinical manifestations of tertiary syphilis vary greatly; however, the most common clinical manifestations include aortitis, gummatous syphilis, and neurosyphilis.

Syphilis remains a public health concern worldwide, with data indicating that the rate of infection is increasing among men who have sex with men (MSM). According to the Centers for Disease Prevention and Control (CDC) 2020 surveillance report, this community accounts for 43% of all cases in the United States [3]. Ocular syphilis, a subtype of neurosyphilis, is broadly defined as an inflammatory response in any ocular structure secondary to *T. pallidum* infection [4]. The condition may develop at any of the four stages of syphilis, and uveitis is the most common manifestation. However, syphilitic uveitis occurs in only 1-2% of all cases of syphilis since the introduction of penicillin [5].

The diagnosis of ocular syphilis relies on a combination of clinical findings and serologic testing, and it is recommended for all patients with syphilis to get tested for human immunodeficiency virus (HIV) co-infection [5]. The first line treatment for syphilis is benzathine penicillin G and evidence for alternate treatments is limited. We present a patient with a medical history of controlled HIV and penicillin allergy who presented to the office with uveitis and elevated rapid plasma reagin (RPR) titers. HIV RNA quantitative polymerase chain reaction (QN PCR) testing was conducted and indicated 21 copies/mL, and the absolute CD4+ cell count was 308 cells/µL. He was subsequently treated with a 14-day course of doxycycline and benzathine penicillin G after desensitization and demonstrated a significant clinical and serologic improvement.

## Case presentation

A 24-year-old male with a medical history of HIV presented to the office with two weeks of progressive left eye pain and worsening blurry vision. At the time of onset, he presented with complaints of eye redness and discharge and was subsequently diagnosed with possible bacterial conjunctivitis and prescribed erythromycin 5 mg/g ophthalmic ointment for 10 days. He reported symptomatic improvement within two days and subsequent discontinuation of the medication. The symptoms returned within seven days accompanied by eye pain and blurry vision. He rated the pain as 9/10 on his left eye and described the quality as a “poking” sensation. He denied any history of accidents or trauma to the eye. The symptoms were associated with a productive cough and increased sinus pressure and drainage. The pain was alleviated by applying warm water compresses and worsened by closing the eye. He previously experienced watery diarrhea, fatigue, and sinus pressure three weeks prior to the onset of the ocular symptoms but reported that he tested negative for coronavirus disease 2019, and his symptoms subsided.

A review of systems was negative for fever, chills, night sweats, headache, nausea, vomiting, eye floaters, sore throat, chest pain, palpitations, shortness of breath, cough, abdominal pain, hematochezia, penile lesions, abnormal penile discharge, dysuria, hematuria, changes in weight, muscle pain, weakness, loss of balance, abnormal bleeding, or rashes. He denied any sick contacts, recent travel, hospitalizations, or emergency department (ED) visits. The patient was compliant with active medications, which included Biktarvy 50-200-25 mg one oral tablet once a day for HIV. The patient reported an allergy to penicillin, which according to him caused a severe adverse reaction of a rash and swelling of the mouth. The patient had no surgical history. He was living with a roommate who had no similar symptoms and reported that he drank “a lot” on social occasions, but was unable to quantify frequency and quantity. He reported intermittent use of marijuana and denied smoking tobacco products. The patient noted that he was not sexually active at the time; however, he reported multiple partners within the last year, all of which were men.

On physical examination, the patient was afebrile, and the remaining vital signs were within normal limits. The patient appeared to be in mild distress secondary to pain but was answering questions appropriately. Cardiac, respiratory, gastrointestinal, and ​​genitourinary examinations were unremarkable. On ocular examination, the left eye was diffusely erythematous with mild purulent discharge. There was extreme photophobia and decreased visual acuity of the left eye with a normal right eye examination. Reflexes were 2+ and strength was 5/5 in the upper and lower extremities, bilaterally, without any focal neurologic deficits. Brudzinski and Kernig’s signs were negative, and there were no additional signs of meningitis. There was no evidence of cervical lymphadenopathy. Skin evaluation was negative for rashes and ulcerative lesions.

A laboratory panel was ordered, including, but not limited to, the findings presented in Table 1, and the patient was advised to go to the ED for urgent ophthalmology evaluation. On ocular examination, the patient’s left eye appeared erythematous, and based on his acute blurry vision and ocular pain, the decision was made to refer him to the ED for further workup and management. He was also advised to seek an ophthalmologist evaluation as soon as possible.

**Table 1:** Hematological, biochemical parameters, and complementary examinations. HSV 1/2 AB (IgM) = herpes simplex virus 1 and 2 immunoglobulin M antibody; HSV 1 AB (IgG) = herpes simplex virus 1 immunoglobulin G antibody; RPR titer = rapid plasma reagin titer; FTA-ABS = fluorescent treponemal antibody absorption test; HIV 1 RNA, QN PCR = human immunodeficiency virus 1 ribonucleic acid, quantitative polymerase chain reaction

Test	Result	Reference
White blood cells	7.9 × 10^3^/μL	3.8–10.8 × 10^3^/μL
Red blood cells	4.79 × 10^7^/μL	4.20–5.80 × 10^7^/μL
Hemoglobin	14.6 g/dL	13.2–17.1 g/dL
Platelet count	324 × 10^9^/L	140–400 × 10^9^/L
Absolute neutrophils	5,364 cells/μL	1,500–7,800 cells/μL
Absolute lymphocytes	1,714 cells/μL	850–3,900 cells/μL
Absolute monocytes	679 cells/μL	200–950 cells/μL
Absolute eosinophils	119 cells/μL	15–500 cells/μL
Absolute basophils	24 cells/μL	0–200 cells/μL
Sodium	135 mmol/L	135–146 mmol/L
Potassium	3.9 mmol/L	3.5–5.3 mmol/L
Calcium	9.7 mg/dL	8.6–10.3 mg/dL
Albumin	4.5 g/dL	3.6–5.1g/dL
Total proteins	8.1 g/dL	6.1–8.1 g/dL
Antinuclear antibody	Negative	-
C-reactive protein	18.8 mg/L	<8.0
Sedimentation rate	122 mm/hour	
HSV 1/2 AB (IgM)	Negative	-
HSV 1 AB (IgG)	28.50	<0.90
RPR titer	1:1,024 (reactive)	Non-reactive
FTA-ABS	Reactive	Non-reactive
Absolute CD4 count	308 cells/µL	500–1,400 cells/µL
HIV 1 RNA, QN PCR	21 copies/mL	<20 copies/mL

At the ED, an orbit computed tomography ruled out any injuries, vascular, or space-occupying lesions. Blood work indicated normal complete blood count (CBC) and comprehensive metabolic panel (CMP) with elevated inflammatory markers (C-reactive protein and sedimentation rate). The fluorescent treponemal antibody absorption test was highly elevated. There was also an increased RPR at 1:1,024, and his herpes simplex virus 1 (HSV1) immunoglobulin G (IgG)-specific antibodies were also elevated. HIV RNA quantitative PCR testing was conducted and indicated 21 copies/mL, and the absolute CD4+ cell count was 308 cells/µL. The patient was diagnosed with presumed ocular syphilis based on erythematous left eye associated with significantly elevated RPR and HIV status. He was prescribed ocular antibiotic drops and oral doxycycline 100 mg BID for 14 days due to penicillin allergies.

A week after the hospital visit, the patient was seen at the clinic for a follow-up consultation. He admitted to not having followed up with the ophthalmologist and noted that his symptoms were alleviating. The patient was subsequently evaluated after completing treatment with doxycycline, and marked improvement in symptoms and examination findings were noted. As a precautionary measure, it was decided to proceed with penicillin desensitization and treatment with intramuscular benzathine penicillin G. After completion of treatment, his ocular examination findings were unremarkable and the patient denied pain, ophthalmalgia, or any other symptoms. Blood tests were ordered and his RPR had significantly decreased from 1:1,024 to 1:32, which is more than a four-fold decrease, which is considered curative.

## Discussion

The diagnosis of ocular syphilis is generally made in patients with serologic evidence of syphilis who present with ocular signs or symptoms, such as eye pain, redness, photophobia, diminished visual acuity, or vision loss [6]. HIV infection is a well-known association, likely due to the shared mode of transmission, and ocular syphilis has been reported as one of the initial indicators of acquired immunodeficiency syndrome. HIV-positive patients tend to have more severe disease as a result of a suppressed immune system as approximately 10% of HIV patients contract syphilis in their lifetime and contract ocular syphilis approximately 50% of the time [7]. Several studies have indicated higher rates of panuveitis, posterior segment, and optic nerve involvement in this population. However, the long-term complications, including vision loss and visual impairment, do not appear to be related to HIV status [8].

In HIV-positive patients with suspected ocular syphilis, it is critical to evaluate for clinical signs indicating central nervous system (CNS) involvement such as altered mental status, headache, cranial nerve deficits, or meningismus, which previous studies have reported in up to 22% of cases. Additionally, serologic studies such as the fluorescent treponemal antibody absorption test (FTA-ABS) should be obtained. In this population, when ocular symptoms and positive FTA-ABS are present, as in this case, patients should be referred to ophthalmology to confirm the diagnosis, and treatment for neurosyphilis should be initiated. The workup also includes a lumbar puncture, which is used to determine if there are abnormalities in the cerebrospinal fluid (CSF) and to establish a baseline if abnormal [6,8,9]. In this case, the patient’s physical examination was unremarkable, except for uveitis, and the patient was referred for ophthalmology evaluation.

It is also essential to consider other differential diagnoses when ocular symptoms are the chief complaint, especially in HIV-positive patients. Some of the most important infectious etiologies include ocular tuberculosis, ocular toxoplasmosis, cytomegalovirus retinitis, herpetic retinitis, and, less commonly, arboviral infections. Non-infectious etiologies include acute angle-closure glaucoma, sarcoidosis, Behçet disease, and, rarely, primary or metastatic neoplastic conditions [8]. In this case, the workup was negative except for highly elevated RPR titers of 1:1,024. Considering these findings with the physical examination, social, and travel history, ocular syphilis was the most likely diagnosis.

The CDC recommends benzathine penicillin G 2.4 million units intramuscular in a single dose as the first-line treatment of syphilis in HIV-positive patients. In patients with penicillin allergy, like in this case, alternate therapies should be considered. Currently, the only alternative treatments the CDC recommends for patients with latent syphilis are doxycycline 100 mg orally twice daily for 28 days or tetracycline 500 mg orally four times daily for 28 days. If compliance is an issue, another option would be to desensitize the patient and then treat with benzathine penicillin G. There are no sufficient studies describing the efficacy of doxycycline or tetracycline as alternative treatments in patients with HIV [10]. In this case, the patient was originally treated with the doxycycline regimen while nontreponemal antibody titers were monitored closely and demonstrated a continual downtrend. The patient was then switched to intramuscular penicillin for 14 days after desensitization. Interestingly, after the treatment course of doxycycline and penicillin, the RPR titers declined from 1:1,024 to 1:32. Treatment response in syphilis management is demonstrated by a >four-fold decrease in non-treponemal antibody titers, which was achieved in our patient, or seroconversion to non-reactive titers [11,12]. The significant decrease in RPR titers observed in our patient, a 32-fold decline, suggests that HIV-positive patients may also respond appropriately to these alternative regimens.

## Conclusions

Our patient initially received a 14-day course of doxycycline for suspected ocular syphilis with subsequent improvement of his symptoms. Even though the efficacy of doxycycline as an alternative treatment for ocular syphilis in patients with HIV/AIDS has not been sufficiently studied, this might suggest that these patients could potentially be good candidates for treatment with this drug. Therefore, this is an important consideration in future HIV-positive patients with syphilis and penicillin allergies. More studies are needed to make these therapies available with evidence-based support.
